# Use of the pectoralis minor and coracoacromial ligament for a biplanar coracoclavicular and acromioclavicular reconstruction: A cadaveric feasibility study

**DOI:** 10.1002/jeo2.70032

**Published:** 2024-10-08

**Authors:** Marco A. Cartaya, Jorge M. Vargas

**Affiliations:** ^1^ Medicine, Orthopaedic and Trauma Department Universidad Finis Terrae Clinica Las Condes Santiago Chile; ^2^ Orthopaedic and Trauma Department Hospital del Trabajador ACHS Santiago Chile

**Keywords:** acromioclavicular dislocation, coracoacromial ligament, pectoralis minor, reconstruction, transfer

## Abstract

**Purpose:**

The aim of this study was to evaluate the feasibility of a novel technique that focuses on vertical and horizontal stabilization of the acromioclavicular joint using two local autologous grafts, the pectoralis minor (Pm) and the coracoacromial ligament (CAL).

**Methods:**

Ten fresh‐frozen shoulder cadaveric pieces were dissected. Length and width of the Pm and CAL were measured in their anatomical position and anatomical variants were noted. The Pm tendon was harvested at the myothendinous junction keeping the insertion at the coracoid process. The CAL was detached from the coracoid process keeping the acromial insertion. The free limbs of both grafts were prepared with the Krackow technique and the Arthrex SpeedWhip technique, respectively. The primary coracoclavicular reduction and fixation were with the button system or with two subcoracoid ultrahigh‐strength suture cerclage through and around the clavicle. The Pm graft was fixed inside a clavicular tunnel by a cortical button and the CAL was transferred and fixed to the lateral clavicle using a knotless anchor or intramedullary when lateral clavicle resection was performed.

**Results:**

The median length of the Pm was 50 mm (interquartile range [IQR]: 50–54), and the median length of the CAL was 36.5 mm (IQR 34–40) which decreased by 15% and 23% once were prepared with the Krackow and Arthrex SpeedWhip techniques to 44.5 mm (IQR: 30–65) and 30 mm (IQR: 22–32), respectively. The diameter of the prepared Pm graft was 5 mm (IQR: 4.5–6) and the CAL graft 5.5 mm (5–6). All grafts were able to reach the fixation points. The procedure was feasible in 100% of the cases.

**Conclusion:**

A biplanar reconstruction using autologous Pm and CAL appears feasible in restoring the acromioclavicular joint stability.

**Level of Evidence:**

Level IV. Basic science, anatomy, cadaveric dissection

**Abbreviations**
AC: acromioclavicular
ACJ: acromioclavicular joint
CAL: coracoacromial ligament
CC: coracoclavicular
CT: conjoined tendon
Pm: pectoralis minor

## INTRODUCTION

Acromioclavicular joint (ACJ) dislocation is a common shoulder injury, especially in young athletes, with an incidence rate of 9.2 per 100,000 person‐years, and it accounts for more than 40% of shoulder injuries in individuals who engage in contact sports [12, 24]. The treatment of ACJ dislocation depends on grade of displacement, severity of symptoms, and the time elapsed since the injury. The cutoff point for defining chronic injury has been debated, traditionally set at 3 weeks after trauma [31], but some studies have proposed 2 [10, 22], 4, or even 6 weeks [27].

When in chronic cases conservative treatment fails, several surgical techniques are available, including anatomical and non‐anatomical repairs and reconstructions. Biological reconstructions are widely used due to the low healing potential of the native coracoclavicular (CC) ligaments in chronic ACJ dislocation [4].

Weaver and Dunn (1972) introduced a non‐anatomical ACJ dislocation reconstruction method, involving lateral clavicle resection and coracoacromial ligament (CAL) transfer as a vertical stabilizer [30]. This technique, modified over time for improved mechanical stability during graft healing, aims to minimize the risk of failure [5]. Mazzoca et al. developed a popular anatomical CC reconstruction using a semitendinosus allograft fixed at the clavicle footprint with two interference screws, yielding biomechanical results comparable to native CC ligaments. Despite superior clinical outcomes compared to the Weaver Dunn procedure [6, 18, 19], this technique poses complications such as donor site morbidity, high cost, receptor rejection, and fractures [8, 17, 28].

Recent interest has focused on reconstructing the acromioclavicular (AC) ligamentous complex. Nakazawa et al. identified the anteroinferior and posterosuperior bands as key components. The posterosuperior band is crucial for resisting horizontal translation, while the anteroinferior band contributes to rotational load resistance [23]. New techniques combining AC and CC repairs or reconstructions aim to enhance clinical outcomes and reduce radiological failure, addressing residual horizontal instability [26, 29].

Monfair et al. studied the anatomical features of the pectoralis minor (Pm) tendon, comparing its biomechanical properties under axial loads to those of native CC ligaments and CAL. Their findings suggest the Pm tendon is a viable local autograft with biomechanical characteristics similar to CAL [20].

Historically, CAL transfer has been explored mainly as a vertical stabilizer in techniques like the Weave–Dunn method. Recent studies by Le Hanneur et al. have investigated its potential as a horizontal stabilizer as well [14, 15].

This study aims to assess the feasibility of using Pm tendon autograft and CAL transfer to reconstruct CC and AC ligament complexes, providing stabilization to the ACJ in two planes using local autologous grafts.

## MATERIALS AND METHODS

### Cadaveric study

A cadaveric dissection was performed on ten pieces of fresh‐frozen shoulders (five men and five women), six left and four right shoulders. Each specimen consisted of a complete cadaveric piece from medial clavicle to humerus midshaft including the entire scapula.

**Figure 1:**
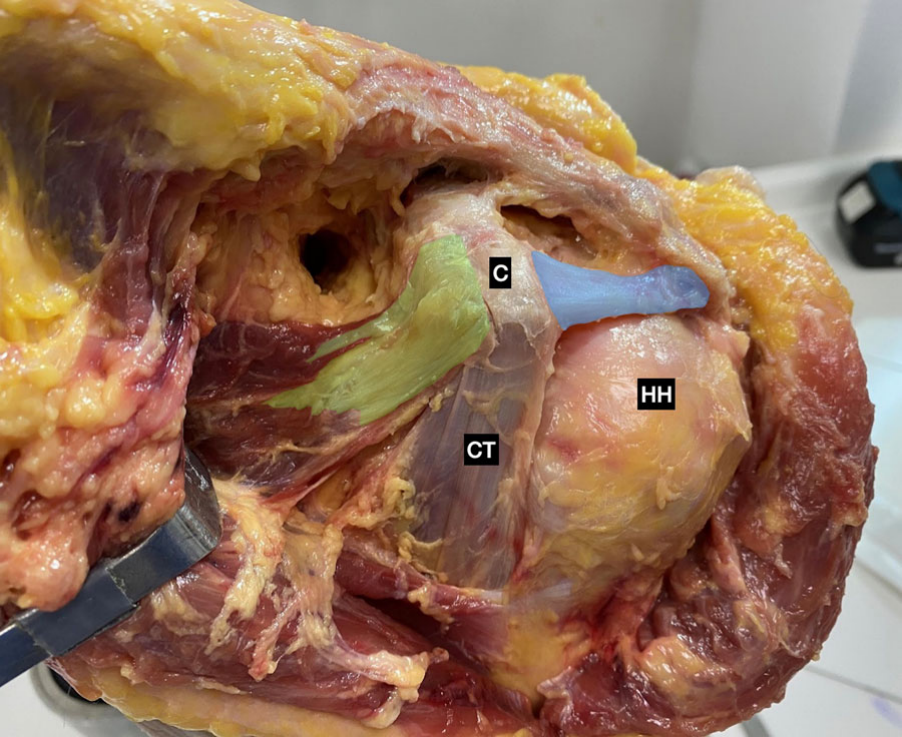
Cadaveric anatomical dissection of a left shoulder: pectoralis minor tendon (highlighted in green), coracoacromial ligament (highlighted in blue). C, coracoid; CT, conjoined tendon; HH, humeral head.

**Figure 2:**
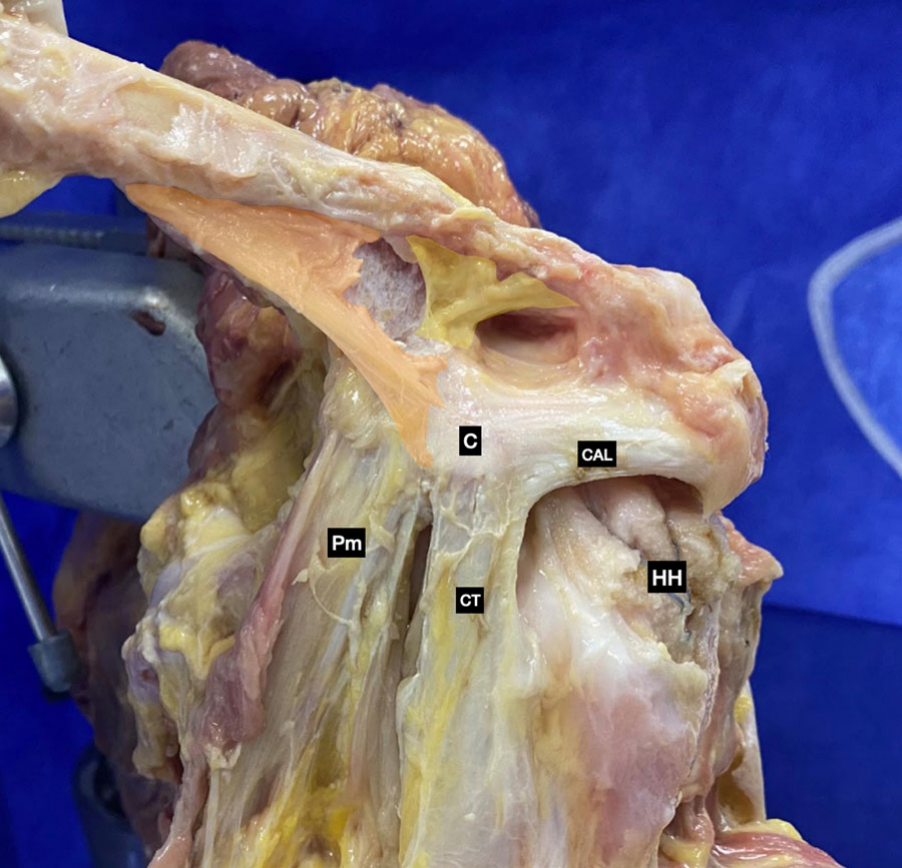
Cadaveric anatomical dissection of a left shoulder showing coracoclavicular ligaments: medial coracoclavicular ligament (highlighted in orange), lateral coracoclavicular ligaments (highlighted in yellow). C, coracoid; CAL, coracoacromial ligament; CT, conjoined tendon; HH, humeral head; Pm, pectoralis minor.

The samples did not present previous injuries or surgeries. After a larger ‘T’ shape skin incision was done, the deltoid, trapezius, and lateral pectoralis major were detached in order to expose the anatomical structures of interest. The Coracoid, CAL, Pm, and conjoined tendon (CT) were properly identified. The medial y lateral CC ligaments and AC ligament complexes were also identified (Figures 1 and 2).

Then the Pm graft was prepared stripping the tendon from the muscle belly using one blade of the Mayo scissor, maintaining the coracoid insertion intact. The length and width of the tendon were measured at the musculotendinous junction and the coracoid enthesis using a metric ruler.

The CAL was identified, and anatomical variables were described. The length was measured at its anterior edge and the width was measured in both insertions. In cases of double band variants measurements were performed in the anterior band only. Additionally, the distance from the ACJ to conoid and trapezoid ligaments was registered.

A Rockwood type V injury was then simulated by transecting the AC and CC ligaments.

### Pm and CAL graft preparation

The previously dissected Pm was tubularized with the Arthrex SpeedWhip technique [32] using FiberLoop® #2 (Arthrex). The CAL was detached from the coracoid, maintaining the acromial insertion, and prepared with Krackow stitches using a FiberWire® #2 (Arthrex). After preparation, the length and diameter of both grafts were measured using an Anterior Cruciate Ligament Graft Sizing Block.

### CC reduction and Pm fixation

Two types of constructs were used: Button system (AC‐Tightrope, Arthrex) or a double ultrahigh‐strength suture cerclage (Fibertape, Arthrex).

For both systems the clavicle was drilled 3.5 cm medial to the ACJ line using a 4.0 mm drill bit for the Pm graft. If necessary, only the inferior cortex of the clavicle was enhanced to match the size of the Pm graft diameter using a flip‐cutter (Arthrex).

**Figure 3:**
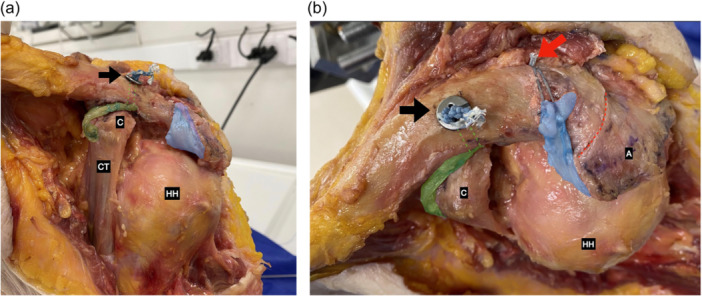
(a) Anterior view of a left shoulder. Biplanar reconstruction with the bottom system. The pectoralis minor tendon graft (highlighted in green) passes through a vertical clavicle tunnel (green dotted line) with a system button (black arrow). The CAL graft (highlighted in blue) was transferred and fixed to the lateral clavicle with a posterior knotless anchor. (b) Top view of a left shoulder. Biplanar reconstruction with bottom system. The pectoralis minor tendon graft (highlighted in green) passes through a vertical clavicle tunnel (green dotted line) with a system button (black arrow). The CAL graft (highlighted in blue) was transferred and fixed to the lateral clavicle with a posterior knotless anchor (red arrow). Acromioclavicular joint (red dotted line). C, coracoid; CAL, coracoacromial ligament; CT, conjoined tendon; HH, humeral head.

When using the button system (AC‐Tightrope®), also the coracoid was drilled with the 4.0 mm drill bit and the inferior button was passed through the clavicle and coracoid. The Pm graft suture strands were then retrieved only through the clavicular tunnel from inferior to superior and added to the superior button before locking the AC‐Tightrope®. The button system was closed and tied in place to achieve the final reduction. The Pm sutures were tied over the clavicular button checking the correct graft tension (Figure 3a,b).

**Figure 4:**
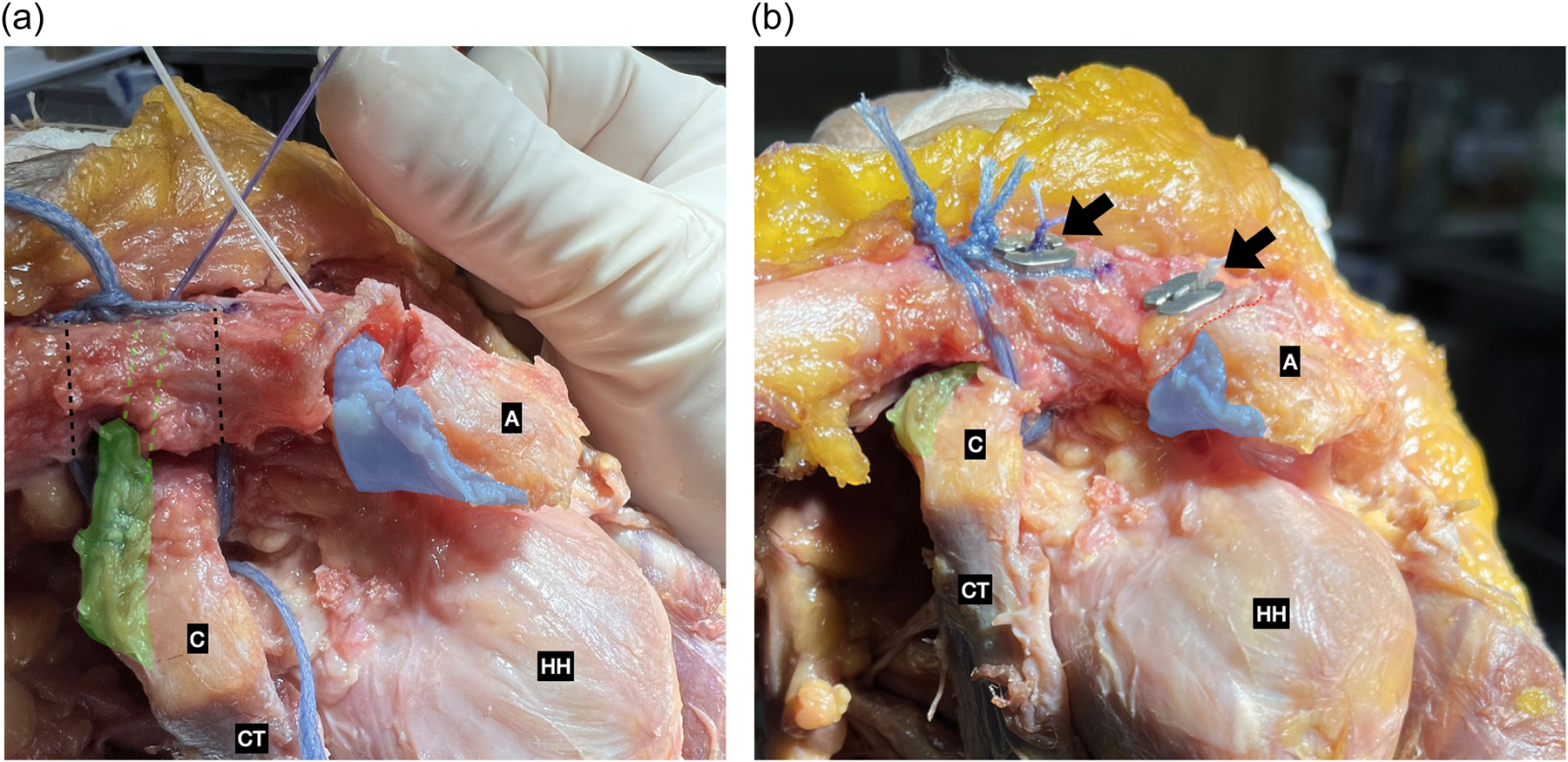
(a) Anterior view of a left shoulder. Biplanar reconstruction with double ultrahigh‐strength suture cerclage. The pectoralis minor tendon graft (highlighted in green) passed through the clavicle in an independent 4.0 mm tunnel (green dotted line) and two 2.5 mm tunnels for double fibertape fixation (black dotted lines) and CAL graft (highlighted in blue) transfer through the lateral clavicle resection. (b) Anterior view of a left shoulder. Biplanar reconstruction with double ultrahigh‐strength suture cerclage. Final construct with the pectoralis minor tendon (highlighted in green) associated with two double fibertape fixation and CAL transfer (highlighted in blue) through lateral clavicle resection. Both grafts are fixed with a superior cortical bottom (black arrows). A, acromion; C, coracoid; CAL, coracoacromial ligament; CT, conjoined tendon; HH, humeral head.

When using the suture cerclage, the first suture passed subcoracoid and periclavicular, while the second suture passed subcoracoid and through two 2.5 mm clavicular tunnels located 10 mm away at each side from the central 4.0 mm tunnel of the Pm graft. The final fixation was achieved with a Nice knot [3] of both sutures above the superior clavicular cortex, while the Pm graft was fixed with a superior cortical button (ABS, Arthrex) (Figure 4a,b).

### CAL augmentation

The CAL graft was fixed with a 2.9 mm Pushlock® anchor (Arthrex) at the union of the posterior ⅓ to the anterior ⅔ of the superior aspect of the lateral clavicle, 10 mm medial to the ACJ. Thus, an anterosuperior reinforcement of the ACJ was obtained by transferring the CAL graft. In cases when distal clavicle resection was performed, the graft was inserted intramedullary and its sutures fixed in the superior cortex with a button in an inverted Weaver Dunn fashion (ABS, Arthrex) [21, 22] (Figures 3a,b and 4a,b).

## RESULTS

The anthropometric measurements are summarized in Table 1.

**Table 1:** Anthropometric measurement of the pectoralis minor and coracoacromial ligament grafts.

	Millimeters^a^
Pectoralis minor	
Length (mm)	50 [40–70]
Length with Krakov (mm)	44.5 [30–65]
Width at the coracoid insertion (mm)	17.5 [13–21]
Width at the myotendinous joint (mm)	18 [17–20]
Width with Krakov (mm)	5 [4–6]
Coracoacromial ligament	
Length at the anterior edge (mm)	36.5 [33–40]
Length with Krakov (mm)	30 [22–32]
Width at the coracoid insertion (mm)	24.5 [13–35]
Width at the acromial insertion (mm)	20.5 [20–24]
Width with Krakov (mm)	5.5 [5–6]

^a^ Median and interquartile range.

The median length of the Pm was 50 mm (interquartile range [IQR]: 50–54), the median width at the coracoid insertion side was 17.5 mm (IQR: 15–20) and 18 mm (IQR: 17–20) at the musculotendinous junction. The median length of the CAL was 36.5 mm (IQR: 34–40) and the median width was 24.5 mm (IQR: 17–28) at the coracoid insertion side.

After preparing the Pm with the SpeedWhip technique, the diameter was 5 mm (IQR: 4.5–6) and the median length was reduced by 5.6 to 44.5 mm (IQR: 40–45). The CAL graft diameter was 5.5 mm (IQR: 5–6) and its length decreased by 6.5 to 30 mm (IQR: 27–30) after preparation.

**Figure 5:**
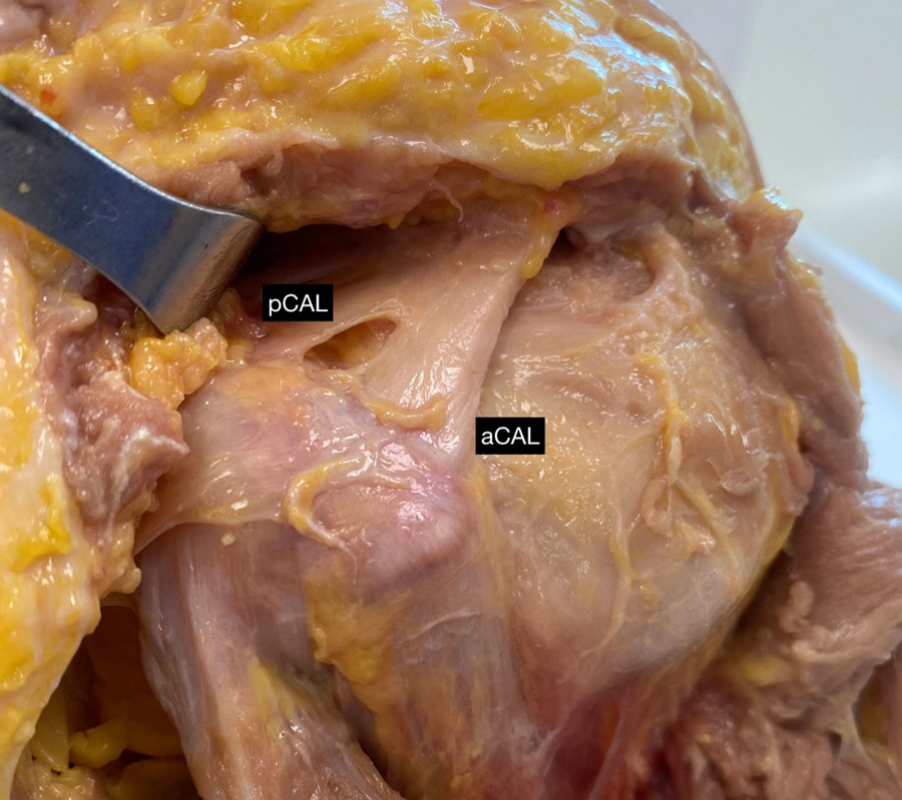
Coracoacromial ligament anatomical variation. aCAL, anterior band; pCAL, posterior band.

It was not found anatomical variables of the Pm. However, in 4 (40%) cadavers the CAL has a double‐band anatomical variation (Figure 5).

The medial CC ligament was identified in all cadaveric samples, posterior to the Pm coracoid enthesis.

The procedure was feasible in 100% of the cases.

## DISCUSSION

This study demonstrates the feasibility of biplanar reconstruction of the stabilizing ligament complex of the ACJ using two local grafts—the Pm and the CAL.

There is no gold standard for the treatment of chronic AC injuries. We introduce a novel technique that eliminates the need for secondary approaches or excessive expenses in allografts, while providing both biological and mechanical components for CC and AC ligaments reconstruction.

Although the Pm tendon has not been previously used as a graft in ACJ reconstructions, it has similar biomechanical properties to the CAL on axial loads [20]. Other advantages of using the Pm tendon are that the healing process of the graft depends only on the clavicular side, sparing the coracoid enthesis of the Pm tendon, and it was long enough to reach the fixation point on the clavicle in all samples. It should be noted that tenotomy of this tendon is one of the treatment alternatives for scapular dyskinesis, a pathology that is highly associated with chronic ACJ dislocations [25]. However, harvesting the tendon could increase the risk of neurovascular injuries when it is prepared by stripping the tendon from the muscle belly due to the proximity between the coracoid process and the brachial plexus [16].

In this study, we did not find anatomical variants of the Pm in 10 cadaveric samples. However, there exists a 10% occurrence of anomalies in the ultrasound regarding the insertion of the Pm tendon [9], without coracoid process enthesis [1]. In such cases, reconstruction of the CC ligaments with the Pm would not be feasible, necessitating the use of alternative techniques such as hemi‐tendon conjoint [14] or allograft.

The advantage of using the CAL as a graft is that it has been used for many years in this type of surgery. It has well‐known anatomic and biomechanical characteristics, as well as the risks associated with its harvest [11]. There is no evidence of contraindication for harvesting the CAL graft except in cases of massive rotator cuff tear to avoid possible anterosuperior migration of the humeral head [2, 13].

The distal clavicle resection in chronic reconstruction is a subject of debate. Grassbaugh reported a 17% increase in the revision rate for AC reconstructions with lateral resection compared to 0% when resection was not performed [7]. Therefore, both scenarios were tested in this present study.

When the distal clavicle was preserved, the length of the CAL graft allowed for anterior augmentation of the ACJ in 100% of cases with an anchor at the junction of the posterior ⅓ and anterior ⅔ of the lateral clavicle, 10 mm medial to the ACJ. The length of the CAL graft did not allow us to reinforce the posterosuperior capsule, which contains the greatest force for posterior translation. However, the reinforcement of the anterosuperior capsule provides rotational stabilization. According to the length and proper ligament release, it allows for greater coverage of the capsule, but it was not described in how many specimens this coverage was achieved.

The insertion point was subjectively chosen as it seemed to have lower posterior translation in manual stress tests performed in the laboratory compared to different CAL graft fixing points.

When lateral clavicle resection was performed, the graft was passed intramedullary with suture fixation in the superior cortex with or without cortical button fixation like others AC reconstructions with CAL for horizontal stabilization [14, 15, 21].

The subsequent steps entail conducting a biomechanical evaluation of this construct to substantiate its mechanical resistance to vertical and horizontal stresses. Moreover, it is imperative to expound upon the technique to determine the optimal approach to execute this surgery and establish potential neurovascular risks associated with harvesting the Pm and CAL, thereby justifying a clinical series. Initially, this technique should be executed as an open surgical procedure, followed by potential implementation of arthroscopic assistance.

We believe that a good treatment for chronic ACJ dislocation is the reconstruction with primary mechanical CC fixation and biological augmentation using vertical and horizontal grafts.

## CONCLUSION

This biplanar AC reconstruction technique utilizing both the Pm tendon and the coracoclavicular ligament autografts has demonstrated feasibility and shows promise as an alternative reconstruction technique for patients with AC dislocations where biological augmentation is necessary.

## AUTHOR CONTRIBUTIONS

Each author fulfils each of the authorship requirements. Marco A. Cartaya conceptualized and designed the study, performed most of the cadaveric dissections, and critically revised the manuscript as submitted; Jorge M. Vargas performed data collection, participated in cadaveric dissections, performed statistical analysis, interpreted data, wrote the paper, and drafted the final manuscript as submitted. All authors read and approved the final manuscript as submitted. The authors have no funding to report.

## CONFLICT OF INTEREST STATEMENT

The authors declare no conflict of interest.

## ETHICS STATEMENT

The Health and Disability Ethics Committee of Hospital del Trabajador deemed that formal ethics approval was not required for this work.

## Data Availability

Data supporting the results of this study are available within the article. Raw data are available from the corresponding author upon reasonable request.
